# Waste-Based Pervious Concrete for Climate-Resilient Pavements

**DOI:** 10.3390/ma11060900

**Published:** 2018-05-27

**Authors:** Hsin-Lung Ho, Ran Huang, Lih-Chuan Hwang, Wei-Ting Lin, Hui-Mi Hsu

**Affiliations:** 1 Institute of Materials Engineering, National Taiwan Ocean University, No. 2, Peining Rd., Keelung 202, Taiwan; hosl4312@ms24.hinet.net; 2 Department of Harbor and River Engineering, National Taiwan Ocean University, No. 2, Peining Rd., Keelung 202, Taiwan; ranhuang1121@gmail.com; 3 Department of Health and Leisure Management, LanYang Institute of Technology, No. 79, Fuxing Rd., Toucheng Township, Yilan 261, Taiwan; hwanglihchuan@gmail.com; 4 Department of Civil Engineering, National Ilan University, No. 1, Sec. 1, Shennong Rd., Yilan 260, Taiwan; hmhsu@niu.edu.tw

**Keywords:** porous materials, co-fired fly ash, permeability, green materials, cementless materials

## Abstract

For the sake of environmental protection and circular economy, cement reduction and cement substitutes have become popular research topics, and the application of green materials has become an important issue in the development of building materials. This study developed green pervious concrete using water-quenched blast-furnace slag (BFS) and co-fired fly ash (CFFA) to replace cement. The objectives of this study were to gauge the feasibility of using a non-cement binder in pervious concrete and identify the optimal binder mix design in terms of compressive strength, permeability, and durability. For filled percentage of voids by cement paste (FPVs) of 70%, 80%, and 90%, which mixed with CFFA and BFS as the binder (40 + 60%, 50 + 50%, and 60 + 40%) to create pervious concrete with no cement. The results indicate that the complete (100%) replacement of cement with CFFA and BFS with no alkaline activator could induce hydration, setting, and hardening. After a curing period of 28 days, the compressive strength with different FPVs could reach approximately 90% that of the control cement specimens. The cementless pervious concrete specimens with BFS:CFFA = 7:3 and FPV = 90% presented better engineering properties and permeability.

## 1. Introduction

Rapid progress in industry and commerce in recent years has brought with it fast and significant growth in inter-city and intra-city transportation. During the process of urbanization, impermeable surfaces such as roads and sidewalks have expanded and taken up a considerable proportion of land area, thereby causing major changes in the regional water cycle. In the past, urban flood control focused on draining away rainwater to prevent overburdening public facilities and flooding low-lying areas in the suburbs during typhoons and torrential rains [1,2,3]. However, it is difficult to expand the underground drainage systems in cities. Furthermore, recent years have seen a rising awareness of water permeability and water retention in cities. Increasing water permeability entails increasing the permeability of urban surfaces [4,5]. If rainwater can reach the earth beneath, the pores in the soil can hold the water, thereby achieving water retention. Adding infiltration systems to urban environments and applying permeable concrete surfaces can also reduce the burden on drainage systems when the rainfall exceeds engineering design standards. When the weather clears, the water can then evaporate into the air, which lowers pavement temperatures, enhances air quality, and improves urban climate cycles [6].

To reduce the CO_2_ emissions produced in the process of manufacturing cement and concrete, the most direct approach is to reduce cement consumption by replacing cement with pozzolanic materials that have the binding properties of cement [7]. The concept of a circular economy has the potential to profoundly change civil engineering. From this perspective, waste and resource management is an important issue. This has led to increased interest in the development of concrete based on waste reuse for environmental preservation and climate resilience [8]. The most common substitutes for cement are the industrial by-products of coal burning and steelmaking, such as fly ash and slag [9]. Development of effective fly ash or slag concrete formulas enables a decrease in the production costs of concrete as well as in environmental impact. This is achieved through energy conservation, carbon reduction, and optimization of waste recycling [10]. Past studies have used water-quenched blast-furnace slag (BFS) directly as a binder with no Portland cement added. However, due to the stability of the crystal structure of water-quenched BFS, the polarity of water alone is not enough to break the chemical bonds within the water-quenched BFS. Ions with stronger polarity are needed to catalyze the hydration of the water-quenched BFS. Currently, the most common approach involves the use of a strong alkaline solution to stimulate the potential activity of the water-quenched BFS so that it displays hydraulic activity [11]. Experiments in recent years have demonstrated that using basic oxygen furnace slag or co-fired fly ash (CFFA) from circulating fluidized bed combustion (CFBC) can serve as the alkaline activator of BFS. Without an additional alkaline activator, the two materials can undergo an autologous alkaline hydration reaction which effectively mimics cement. On the whole, the resulting cement alternative can only achieve approximately 70% to 80% of the compression strength of pure cement mortar; however, it performs well in comparison to other cementless composite materials [12,13].

The primary industrial product of CFBC is CFFA. Unlike the fly ash by-product of typical coal combustion, CFFA contains large amounts of f-CaO and SO_3_. Furthermore, spherical particles are rare in CFFA because the temperatures in a CFBC furnace (800–900 °C) are generally lower than those in a coal combustion furnace (1300–1500 °C). The high f-CaO content in CFFA causes it to react violently with water and derive self-cementing properties [14], which makes CFFA a feasible substitute for cement. The small number of studies that partially replaced cement with CFFA found that it generally reduces the compressive strength of the cementitious material, increases flowability, and delays the setting time [15,16,17]. Based on existing research, CFFA does not yet meet the construction regulations reached by original fly ash. However, most studies indicate that CFFA can pass the majority of regulations associated with cement and construction as a recyclable green material [18]. The composition and properties of fly ash are determined by the type and source of the fuels used and the combustion conditions of the furnace. Different fuels and sintering temperatures result in varying industry by-products [19]. CFBC temperatures are generally lower, so CFFA rarely reaches a molten state. The by-products mainly comprise crystals, and the trace metals in the fuels are generally released during combustion in a volatile state or form crystalline compounds on the surfaces of fly ash particles [20]. As a result, CFBC residues are strong alkalis and contain several trace metals that may have an adverse impact on the environment. Further analysis is needed to understand the chemical properties of these industrial by-products. The CaO and CaSO_2_ in CFFA are prone to the formation of hydration products such as calcium hydroxide and ettringite. The calcium hydroxide or calcium sulfate that exists within concrete structures may engage in delayed reactions to water and thereby create expansion cracks [20]. Relevant research indicates that CFFA from CFBC may have some influence on the strength of Portland cement (when cement replacements are lower than 20%). If CFFA content exceeds 20%, then the strength of the concrete will decline significantly. When 10% CFFA is added to a concrete specimen, the amount of water must be increased from 1.8% to 3.2% to attain the desired consistency. The increased f-CaO content in CFFA means increasing the water content, and the setting time shortens as the CFFA content increases. If the SO_3_ and f-CaO contents in concrete specimens are controlled at 4.48% and 3.00%, respectively, the volume stability of the cement is acceptable. Furthermore, the primary hydration products of cement and CFFA include C-S-H, ettringite, and other silicate products [21].

Pervious concrete is a porous composite material that is also referred to as no-fines (no fine aggregates) concrete. It contains a narrow size distribution of coarse aggregates, little or no fine aggregates, and a small amount of cement paste that does not fill the voids among the coarse particles [22,23]. Lightweight and porous, pervious concrete has better water permeability than does normal concrete [24]. However, its high porosity also means that it has lower mechanical strength than does normal concrete; its compressive strength ranges from 1.5 to 14.0 MPa [25] and from 2.8 to 28.0 MPa by ACI 522 [26]. However, pervious concrete can be applied to permeable pavement for water permeability and retention. It has the added benefits of making urban environments more appealing to the eye and mitigating the heat island effect. The voids in pervious concrete can be filled with soil to allow plants to grow, maintain natural ecological functions, and green the environment. Such pavement can be used in paths for pedestrians or streets with light traffic.

This study completely replaced cement with a mix of CFFA and water-quenched BFS, and formulated mix designs according to ACI 522 to create pervious concrete with no cement. We then tested the engineering properties of the resulting specimens, including compressive strength and permeability, to determine whether they meet the basic requirements of general pervious concrete. We chose single-size coarse aggregates (19~25 mm). For the filled percentage of voids by cement paste (FPVs), we experimented with 70%, 80%, and 90%. We chose a water-binder ratio of 0.35 and different mixtures of CFFA and water-quenched BFS as the binder (40 + 60%, 50 + 50%, and 60 + 40%) to create green pervious concrete with no cement. We tested its engineering properties (pH measurement, compressive strength, splitting strength, permeability, water absorption, sulphate resistance, and unit weight) and further observed the microstructures and chemical composition of the binders using X-ray diffraction (XRD) and mercury intrusion porosimetry (MIP).

## 2. Materials and Methods

### 2.1. Materials

In this study, we used Type-I Portland cement from the Taiwan Cement Corporation (Cement industry, Taipei, Taiwan), which has a fineness of 3310 cm^2^/g and specific gravity of 3.15. Its primary components were analyzed according to ASTM C114 standards and include tricalcium silicate (C_3_S, 49%), dicalcium silicate (C_2_S, 21%), tricalcium aluminate (C_3_A, 7.9%), and tetracalcium aluminoferrite (C_4_AF, 9.4%), as shown in Table 1.

The CFFA used in this study is unlike normal fly ash; in appearance, it is a black powder. It is a by-product of burning coal as the fuel with other general industrial waste (such as pulp sludge, waste wood, and waste tires). With properties similar to original fly ash, CFFA should be able to serve as an auxiliary binder or filler in cementitious composite materials. Chemical analysis shows that CaO content >10% and that SiO_2_ + Al_2_O_3_ + Fe_2_O_3_ content >50%. CFFA had a fineness of 2800 cm^2^/g and specific gravity of 2.73. According to ASTM C618, it meets the designations of Class C fly ash, the differences being that it has a sulfide content greater than 5% and that its activity indices at 7 days and 28 days are higher than the 75% stipulated by ASTM C311. These results demonstrate that the CFFA has pozzolanic effects. Table 1 presents its physical and chemical properties.

The water-quenched BFS used in this study had a fineness of 5860 cm^2^/g and specific gravity of 2.88. Its physical and chemical properties meet the requirements of ASTM C989 as shown in Table 1.

The coarse aggregates comprised gravel processed from natural pebbles mined from the middle and upper sections of the Lanyang River. Due to the needs of the experiment, we employed single-size aggregates (19~25 mm). The specific gravity, water absorption rate, and dry unit weight of the coarse aggregates were 2.68, 0.69%, and 1619 kg/m^3^, respectively.

To retain water, decelerate setting, maintain thickness, and increase adhesiveness in the slurry, we added a thickener containing cellulose ether, a natural-thickening modified starch, and a small amount of surfactant. The cellulose ether was 300HYP4 of Methyl Hydroxy Ethyl Cellulose and the weight added was 0.5% of the weight of binder as per ASTM C91 standards.

Superplasticizers are a type of polymer that can be adsorbed by the surface of the particles to prevent cement particles from clustering in the water, thereby ensuring that the water achieves its lubricating effect. Using superplasticizers enhances the workability of fresh concrete with low water-cement ratios, reduces the amount of water needed, and increases concrete strength. High-strength superplasticizers can disperse cement particles to maximize flowability. In this study, it used the HP 100 fluidizer, which is a high-performance superplasticizer comprising mainly polycarboxylates. It has good hydrophilicity and is mixed with water before use. Its chlorine content is lower than 1% of its own weight in accordance with the Japanese Industrial Standards (JIS).

### 2.2. Mix Designs

The mix designs of the concrete specimens in this study were made in accordance with the methods used in ACI 211.1. Absolute volume was used in the calculations, and the specimens were numbered using two letters and numbers to indicate the pozzolanic material ratio (BFS:CFFA) and FPVs. Single-size aggregates (19 mm~25 mm) were used in the mix designs and the aggregates comprised of gravel processed from natural river pebbles. We experimented on three mix ratios for BFS and CFFA: 50% BFS with 50% CFFA, 60% BFS with 40% CFFA, and 70% BFS with 30% CFFA, and there were three settings for FPVs: 70%, 80%, and 90%. The pozzolanic material was either cement, indicated using “C”, or a mix of BFS and CFFA (e.g., “6” means 60% BFS and 40% CFFA). Finally, the FPV settings of the mix designs were A: 70%, B: 80%, and C: 90%. The water-cementitious ratio was fixed at 0.35. Table 2 presents the mix designs examined in this study.

The experiment focused on fresh concrete properties, hardened properties, durability properties, and microscopic analysis. The outer dimensions were divided into three conditions: ϕ10 × 20, fragments, and powders that can pass a #200 mesh (0.075 mm). The measurement results are presented as the mean of the three specimens in each group for all mixtures. The coefficient of variation was controlled to less than 10%.

### 2.3. Test Methods

In accordance with ASTM E70, pH measurement was performed on freshly mixed concrete using an electronic pH meter.

Unit weight was measured in accordance with ASTM D6023. The concrete was rodded in three layers with 25 times per layer. For the bottom layer, the rod penetrated nearly full depth into the concrete but did not strike the bottom of the measure. The rodding strokes were evenly distributed over the surface of the layer. For the two upper layers, the rod penetrated approximately 25 mm into the layer below with each stroke. After each layer was rodded, the sides of the measure were lightly tapped 10 to 15 times with a rubber mallet to close any voids left by the tamping rod and release any bubbles of air in the concrete. The top surface of the concrete was then layered, and the net weight of the concrete within the measure was weighted. Dividing the net weight by the volume of the measure then produced the unit weight of the concrete.

We referred to ASTM C39 when performing the compressive strength test. Freshly mixed concrete was poured into a ϕ10 × 20 mold and cured for 24 ± 0.5 h in the mold before demolding. The specimens were cured in a curing tank containing saturated lime water and then retrieved for compressive strength testing on the designated days (7, 14, and 28 days). The compressive strength tests were conducted while the specimens were still wet. Any foreign matter on the contact surfaces of the specimens and the testing machine was removed, and the central axes of the specimens had to line up with that of the round base. Uniaxial load tests were performed at a compression velocity of 3.5 kgf/cm^2^, and the compressive strengths of the specimens upon failure were recorded.

For the splitting strength test, we referred to ASTM C496. The purpose of the splitting strength test is to test the tensile strength of the pervious concrete. Testing was performed once the specimens were cured for the designated number of days (7, 14, and 28 days). The splitting strength tests were conducted while the specimens were still wet. The specimens were aligned to the fixture and placed in smoothly. Force was then applied along the length of the specimens, and the splitting strengths of the specimens upon failure were recorded.

Absorption was measured in accordance with ASTM 642. First, ϕ10 × 20 cm cylindrical specimens were cured until the designated 28 days and then dried in an oven at 100 to 110 °C for 24 h. After being removed from the oven, they stood at room temperature to cool. Once their temperatures fell to between 20 and 25 °C, the specimens were weighed and then completely immersed in water for a total of 48 h. At 24 h and 48 h, their surface-dry weights were measured. If the difference between the two measurements was less than 0.5%, then the specimens were considered to be in saturated state. The weights were then recorded and substituted into the formula below to calculate the water absorption of the specimens.

(1) $$\mathrm{Absorption}\;\mathrm{rate}\;(\%)\;=\;\frac{B-A}{A}\times 100\%$$

where *A* is weight of oven-dried sample (g), *B* is weight of saturated specimen (g).

For the sulfate soundness test, we referred to ASTM C88. When the concrete is exposed to a harsh environment, such as harmful chemical substances in the soil or water, the concrete can easily expand and crack, which reduces its strength and shortens its service life. The corrosive environments formed by sulfates generally involve multiple types of salts co-existing in groundwater or seawater. These salts do not exist in simple forms and can only infiltrate concrete in solution form. In addition, they must exceed a certain amount to be hazardous to concrete. This sulfate soundness test determines the soundness of concrete aggregates under the effects of weathering and simulates salt infiltrating the pores of the concrete and corroding the interior. When the specimens are again immersed in a saturated sodium sulfate solution, the interior fills with liquid that simulates the influence of freezing and expanding water on concrete. The simulation conditions of the test environment are sufficient to allow aggregate soundness information through repeated immersions in saturated sulfuric acid. The test process involves placing the specimens in a saturated sodium sulfate solution for 24 h and then drying them in an oven at 110 ± 6 °C for 24 h for the specimens to be partially or completely dried. After one round, the specimens are weighed and five rounds were conducted in this study.

The permeability test was conducted based on the Pavement Testing Manual published by the Japan Road Association [27]. The constant head permeability test was used for calculations, and a permeability tester was used the measure the permeability coefficient of ϕ10 × 20 cm cylindrical specimens. The formula for permeability coefficient K is as follows:

K = QL/AHΔt(2)

where K is permeability coefficient (cm/s), Q is flow (mL), L is specimen thickness (cm), A is permeable surface area of specimen (cm^2^), H is height of water head (cm), Δt is the actual time from t_1_ to t_0_.

The MIP test was conducted in accordance with ASTM D4404. For the testing instrument, we employed the AutoPore IV 9500 mercury porosimeter from the Micromeritics Instrument Corporation (Micromeritics China, Beijing, China) which can measure the diameter of pores within specimens and calculate their cumulative total volume. Pores with a diameter smaller than 10 nm are gel pores, while those with a diameter greater than 10 nm are capillary pores. Based on the diameters and cumulative total volume of the pores, we can calculate the gel pore volume and capillary pore volume in the specimens. Both pore size and volume influence concrete performance. The volume distributions derived from the test can promote understanding on the properties of the concrete. During the tests, test tubes are placed in a high-pressure chamber, and the pressure is increased to 33,000 psi. Pressure, pore diameters, the increase in pore volume, and the cumulative pore volume can be calculated based on the external pressure applied and the amount of mercury forced into the specimen. Once the pressure reaches 33,000 psi, the chamber is depressurized until it reaches atmospheric pressure. Then, the test is completed.

XRD is a non-destructive analysis technique that is used to detect the properties of crystalline materials. It enables the analysis of structure, phase, preferred orientation (texture), and other structural parameters such as average particle size, crystallinity, tension, and crystal defects. For XRD, it used the Siemens D5000 X-ray diffractometer (Siemens, Aubrey, TX, USA), which can analyze the chemical composition of crystalline materials. As cement-based materials are compounds with multiple elements, their powders can be analyzed using XRD to identify their chemical composition.

## 3. Results and Discussion

### 3.1. pH Measurement

In the 12 mix designs, A, B, and C indicate different FPVs, which merely means different amounts of slurry and does not affect the pH values. Thus, after eliminating the FPV condition, only specimens C, 50% BFS, 60% BFS, and 70% BFS remained, and their pH values were 12.3, 12.1, 12.2, and 12.3, respectively. The three experiment groups differed little from the control groups containing normal cement. However, the experiment groups show that as the proportion of CFFA declined from 50% to 30%, the pH value gradually approached the values of the control group (12.3). On the whole, the pH values of the mortars containing various proportions of CFFA and BFS and no cement were close to that of the mortar containing only cement. From the previous study [11,12], we can see that the CFFA and BFS in the experiment groups contain compounds such as Ca(OH)_2_, CaO, CaSO_4_, and SiO_2_, among which CaO and CaSO_4_ engage in hydration reactions with water:

CaSO_4_ + 2H_2_O → CaSO_4_∙2H_2_O, (3)

CaO + H_2_O → Ca(OH)_2_, (4)

The Ca(OH)_2_ continues to react with SiO_2_, thereby producing C-S-H gel. As a result, the pH values of the solutions in the pores remain highly alkaline as they do in the control groups, thereby preserving the gelability of cementless composite materials.

### 3.2. Unit Weight

**Figure 1:**
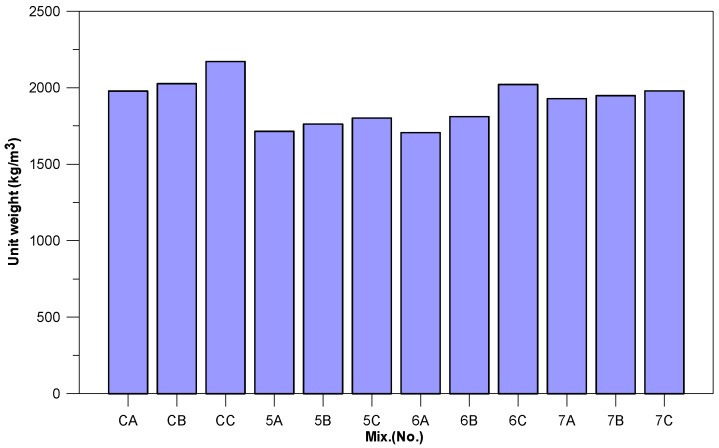
Unit weight of specimens.

Comparison of the specimens with different slurry proportions (50% BFS, 60% BFS, and 70% BFS) and the control groups (C) reveal that the specific weight of the material (cement > BFS > CFFA) exerts significant influence on the unit weight of the concrete as illustrated in Figure 1. Further comparison of the specimens with the same BFS-CFFA ratio and the control groups showed that the unit weights of Specimens 5A, 5B, and 5C only reached 83~86% that of the control groups; those of Specimens 6A, 6B, and 6C only reached 86~93% that of the control groups, and those of specimens 7A, 7B, and 7C reached approximately 91~98% that of the control groups. The main reason is the replacement of cement with CFFA and BFS, which have lower specific gravity than cement (3.15). Thus, when the cement in pervious concrete is completely replaced, the cementless pervious concrete will display a lower unit weight than normal pervious concrete containing cement. On the whole, a BFS-CFFA mix ratio of 7:3 resulted in unit weights closest to that of the control groups, and as the FPV increased from 70% to 90%, the amount of slurry as well as the unit weight increased. The unit weights of most of the specimens fell within the acceptable range for pervious concrete, which is 1400 kg/m^3^ to 1900 kg/m^3^. According to ASTM C330, the unit weight of lightweight concrete must be below 1840 kg/m^3^. Thus, Specimens 5A, 5B, 5C, 6A, and 6B also meet the requirements for lightweight concrete.

### 3.3. Compressive Strength

**Figure 2:**
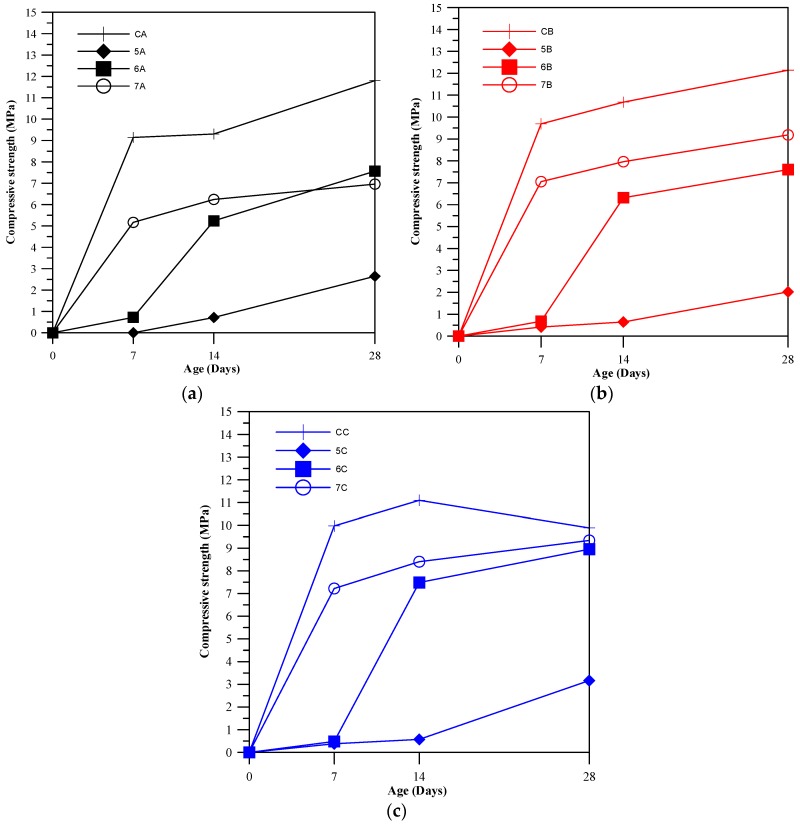
Comparison of compressive strength as: (**a**) FPV of 70%; (**b**) FPV of 80%; (**c**) FPV of 90%.

Figure 2 displays the compressive strength results of the pervious concrete specimens for which the coefficient of variation was controlled to less than 10%. Among the different BFS-CFFA ratios, 7:3 produced the highest compressive strength. After 14 days, the CaO in the CFFA had not sufficiently reacted with the water to produce Ca(OH)_2_, and then needed more time to act that the Ca(OH)_2_ and SiO_2_ in the CFFA and BFS react with Al_2_O_3_ and produce C-S-H or C-A-S-H gel. We speculate that the pozzolanic reactions among the aggregates in the specimens peak at 28 days. At the same time, the FPV influences compressive strength, the two presenting a directly proportional relationship. On the whole, the control groups presented significantly greater compressive strength than the specimens in the experiment groups, regardless of the BFS-CFFA ratio. However, the specimens in the experiment groups still have adequate strength to serve as the materials of waterworks structures or pervious backfill materials that do not need to be compacted, which is 1.5 to 14 MPa [25]. At 28 days their strength is enough for pervious concrete in practice. The specimens with a BFS-CFFA ratio of 7:3 could reach approximately 90% of the compressive strength of the control groups. This is therefore the optimal ratio for green and cementless materials. The compressive strength of pervious concrete relies on the stacked coarse aggregates in the concrete as well as the bonding force between slurry and aggregate. Thus, a higher FPV means a larger amount of slurry used and thus greater compressive strength. The primary components of CFFA (Ca(OH)_2_, CaCO_3_, and CaO) have poorer crystal strength and density than those of cement (C_3_S, C_2_S, C_3_A, and C_4_AF). Consequently, replacing more cement with CFFA will inevitably reduce the amount of slurry and the compressive strength of the resulting specimen. Thus, the cementless specimens all had slightly lower compressive strength than the control groups (pervious concrete packed with cement slurry). In contrast, CFFA contains about 7.36% of SO_3_ and a small amount of gypsum, which react with the calcium aluminate salts in the BFS (such as C_3_A and C_4_AF) to form ettringite (AFt). This product improves the crystallinity and density of C-S-H gel and produces viscous hardening behavior in the cementless slurry.

### 3.4. Splitting Strength

**Figure 3:**
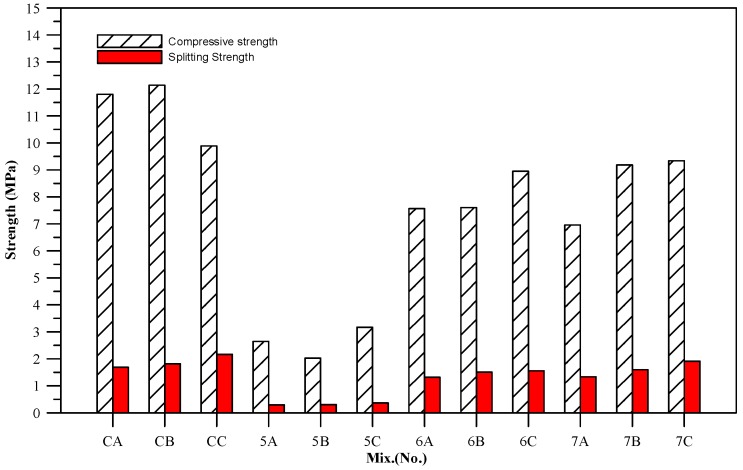
Comparison of compressive strength and splitting strength.

With the different FPVs in Figure 3, the splitting strength of the specimens amounted to roughly 11~22% of their compressive strength. As pervious concrete presents lower compressive strength, the range of the splitting strength-compressive strength ratio in pervious concrete is wider than that in normal concrete, which is approximately 10~15%. The specimens of BFS-CFFA ratios 7:3 (7 groups) displayed better performance than the 5 groups and 6 groups specimens and produced test results that were closer to those of the control groups.

### 3.5. Absorption of the Cementless Pastes

**Figure 4:**
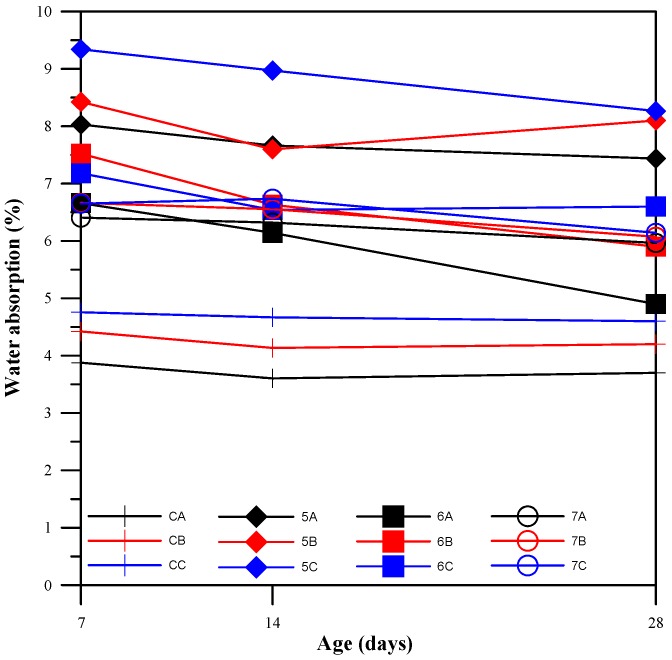
Absorption rates of specimens.

**Figure 5:**
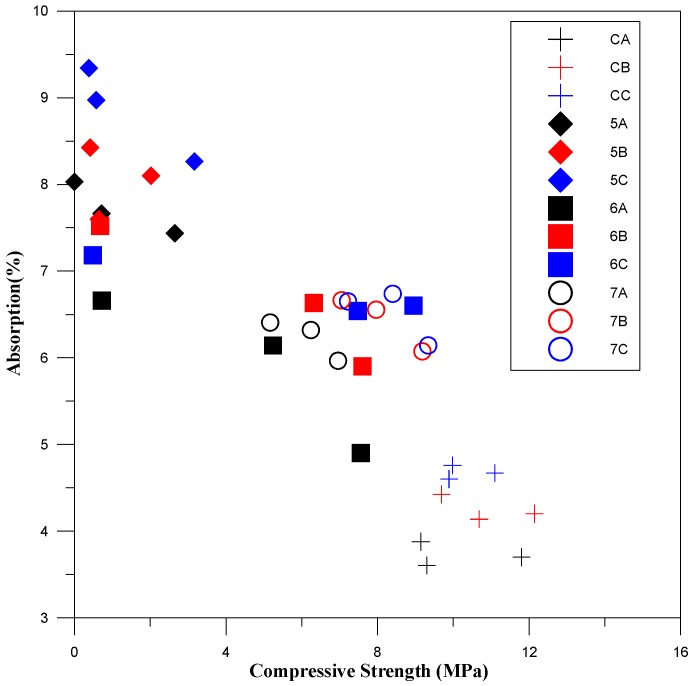
Distributions of absorption rates and compressive strength.

The results for water absorption rate are presented in Figure 4. Specifically, we focus on the absorption of the blended paste within the pervious concrete. Initially, the pervious concrete specimens in the experiment groups required more water than the control groups, which is consistent with a previous study [28]. The water absorption rate test offers a rough estimate of specimen porosity, which also indirectly impacts compressive strength. Figure 5 shows that the specimens of BFS-CFFA ratios 5:5, which have relatively lower compressive strength, have higher water absorption rates. Thus, we can establish that a negative correlation exists between compressive strength and water absorption rate. The compressive strength trends in the experiment and control groups can therefore be estimated based on the results of absorption tests. However, as the curing period increased, the water absorption rates gradually converged, which shows that the hydration reaction within the pozzolanic materials was slowing down and that late strength was coming in. The products from the continuing reactions of the CFFA and BFS gradually filled in the pores, thereby reducing internal porosity and the water absorption rate. At 28 days, the water absorption rates of the specimens in the experiment groups approached those of the control groups.

### 3.6. Sulfate Resistance

The purpose of the sulfate resistance test is to gauge the resistance of cement-based composite materials against sulfates. The two most common types of sulfate corrosion are as follows:

(a) Groundwater contains natural sulfates such as sodium sulfate (Na_2_SO_4_), potassium sulfate (K_2_SO_4_), magnesium sulfate (MgSO_4_), and calcium sulfate (CaSO_4_). Excessive contents of these sulfates mean excessive contents in the soil as well, which results in soil acidification.
(b) Sulfates are one of the main components of seawater. Thus, cement-based materials applied to harbor engineering or marine environments are submerged in seawater for long periods of time and are easily corroded by sulfates in the seawater.

In view of the above, sulfate corrosion is a common problem for structures both on land and in the sea, so sulfate resistance evaluations are extremely important.

**Figure 6:**
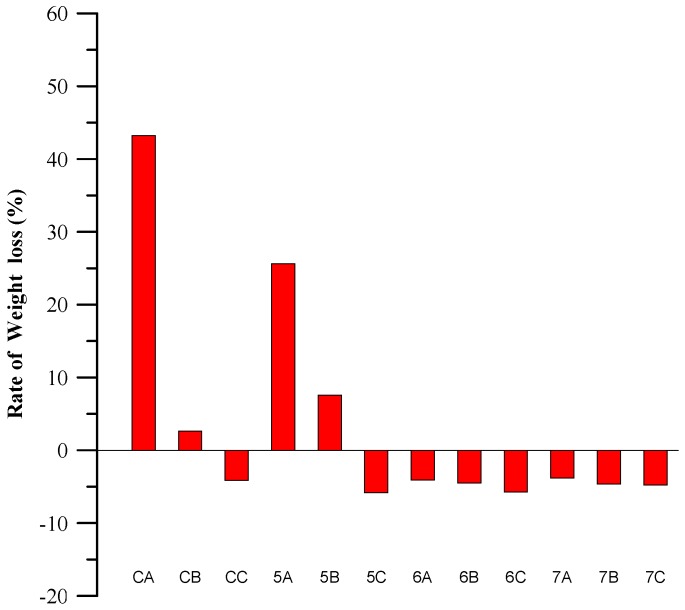
Weight loss statistics of specimens.

**Figure 7:**
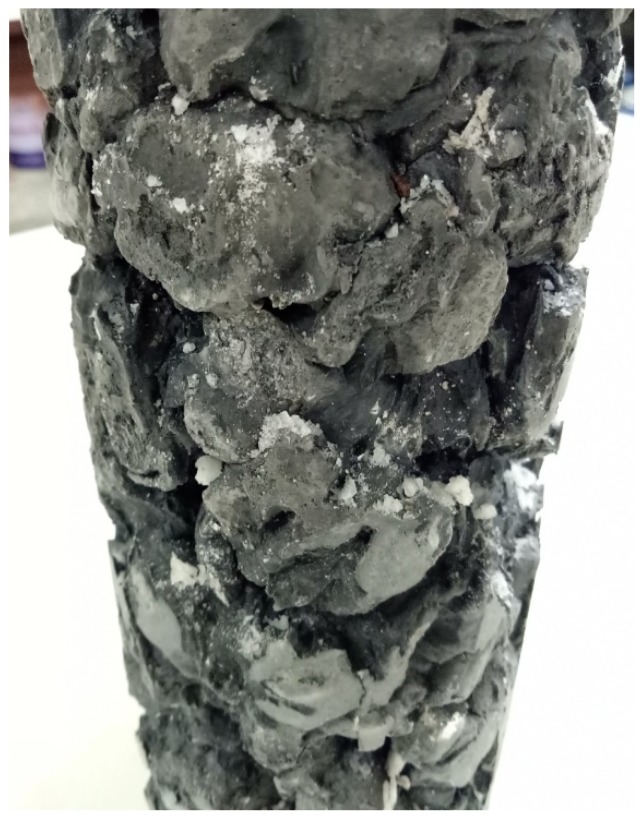
Crystals produced on surface of cementless specimen.

The basic principle of sulfate corrosion is that the sulfate ion (SO_4_^2−^) reacts with the monosulfoaluminate in cement-based materials and causes local expansion in slurry, which leads to internal stress and cracking that affects the compressive strength and durability of the concrete. Completely replacing cement with a mix of BFS and CFFA significantly reduces the C_3_A content. A higher proportion of BFS, in particular, indirectly enhances the sulfate resistance of concrete. As shown in Figure 6, a higher percentage of BFS results in less weight loss or even negative weight loss, which means that the specimens actually gain weight from ettringite crystals attaching themselves to the specimens while they are immersed in the sulfate solution. Therefore, replacing cement with a higher proportion of BFS means greater sulfate resistance. Furthermore, the main crystals in CFFA are Ca(OH)_2_ and CaO. When specimens are immersed in the sodium sulfate solution, sulfate ions (SO_4_^2−^) infiltrate the specimens via pores and cracks and react with Ca(OH)_2_ and CaO that have not yet been consumed. When the specimens are placed in the oven, the sulfate and high-temperature environment may then produce new crystals within the specimens such as the white crystals in Figure 7. This is why the cementless specimens may actually gain weight.

Furthermore, as the FPV increases, the specimens become denser and are less susceptible to sulfate corrosion. Based on the figure, we can see that the 6 groups (40% CFFA and 60% BFS) and 7 groups (30% CFFA and 70% BFS) specimens, which contain more BFS, presented the highest chemical resistance. The main reason is that CFFA contains Ca(OH)_2_ and SO_3_, which react with the C_3_A in the BFS to respectively form ettringite (AFt) and gypsum (CaSO_4_). As the primary corrosion target of sulfate is monosulfoaluminate (AFm), these two factors (Ca(OH)_2_ and SO_3_) can enhance the sulfate resistance of cementless pervious concrete specimens.

### 3.7. Permeability

**Figure 8:**
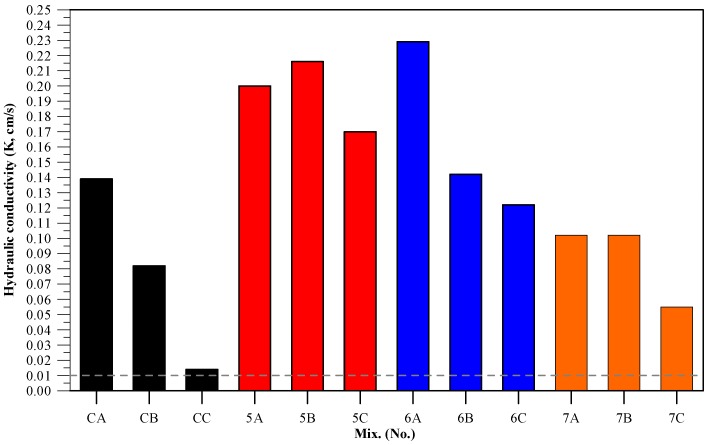
Permeability coefficients of specimens.

Pervious concrete does not contain any fine aggregates that can fill in pores, so aggregate stacks are not dense. A higher FPV means that more of the pores have been replaced with slurry, which in turn means lower permeability. Permeable surface standards recommend that the permeability coefficient of pervious concrete be no less than 1 × 10^−2^ cm/sec. A look at Figure 8 shows that all of the specimens met this requirement. Furthermore, the cementless specimens presented higher permeability coefficients than the control groups. A comparison with the compressive strength results shows that lower compressive strength means a higher permeability coefficient. It speculated that using CFFA and BFS results in greater permeability, and at the same time, an indirect relationship exists between porosity and compressive strength. Moreover, comparison of the specimens with the same type of slurry revealed that as the FPVs increased from 70% to 90%, fewer paths were available to the water, thereby reducing permeability. This is natural because when the filled percentage of voids increased, more voids were filled by binder and consequently the water permeability decreased. This is mainly the function of interconnected filled void content for pervious concrete.

### 3.8. Pore Structure of the Cementless Pastes

**Figure 9:**
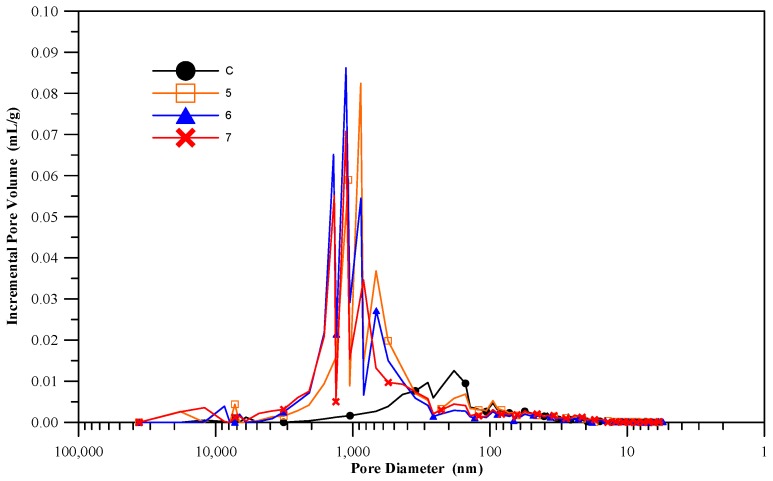
Pore diameter distribution.

**Figure 10:**
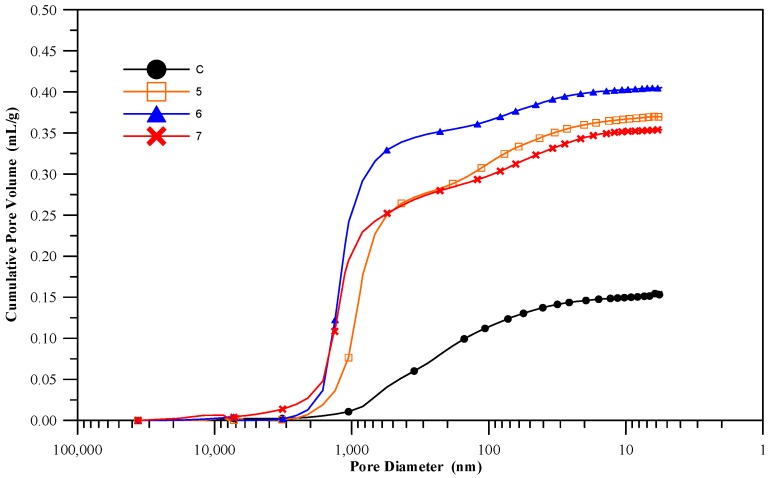
Cumulative total pore volume.

Pore structure is a major factor that influences the permeability and durability of materials. Literature indicates that the pores in concrete can be divided into compaction pores, entrained air, capillary pores, and gel pores, the latter two being smaller and accounting for the majority [29]. The types that have the most impact on concrete durability are entrained air, capillary pores, and gel pores, their diameter ranges being >10,000 nm, 10~10,000 nm, and <10 nm. The diameters of the pores in the control specimens (cement pastes) containing cement were approximately from 100 to 1000 nm (Figure 9), while those in the cementless specimens ranged from 600 to 2000 nm. Most of the pores within this diameter range are capillary pores. The pores within the C group specimens were significantly smaller and presented a wider diameter range, while those in the 5 groups, 6 groups, and 7 groups specimens were much larger and presented a narrower diameter range. The comparison of cumulative total pore volume in Figure 10 shows that the specimens in the control group are significantly more stable trend (lower pore volume) than those in the experiment groups. The total pore volumes in the latter were more than twice as much as those in the former. This may be due to the delayed ettringite formation induced by the C-A-H and sulfides, which cause structural expansion. In addition, the pozzolan hydration reactions in the cementless specimens may have been slower because the specimens contained more water that did not engage in hydration reactions than the cement specimens did, which also left more pores in the specimens after the slurry hardened. CFFA and BFS also absorb more water than cement, so when more CFFA replaces cement, the water-binder ratio will decrease slightly, thereby affecting the workability of the mortar. The air bubbles within the slurry are more difficult to expel via tamping and vibration, which increases the porosity in the specimens and is likely why the cementless materials have higher porosity. The MIP test results can also serve as verification data for the water absorption test.

### 3.9. XRD of the Cementless Pastes

**Figure 11:**
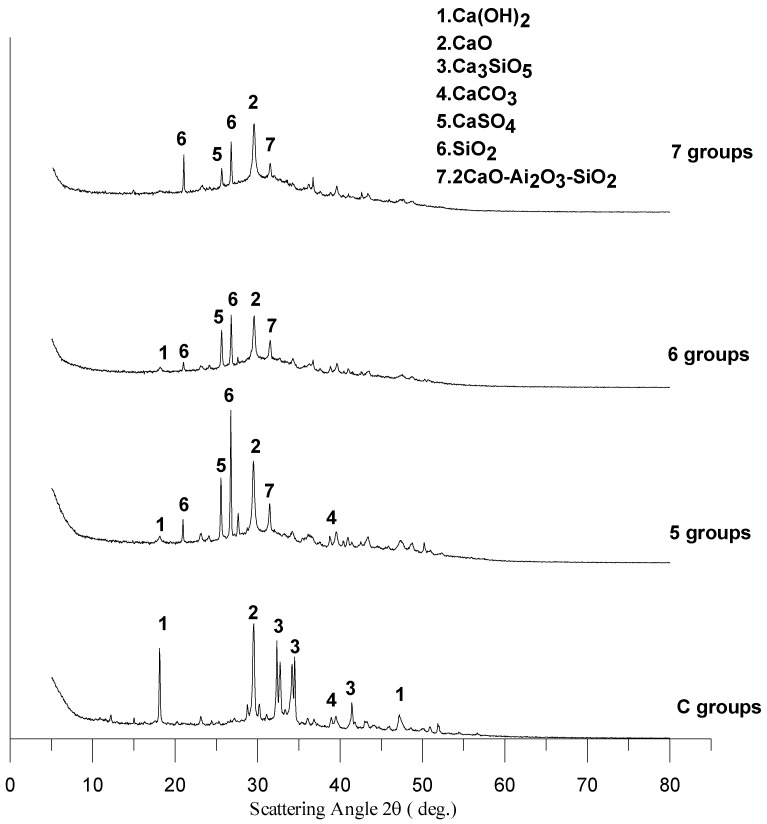
XRD patterns at 28 days.

Cement-based composite materials comprise a number of elements. XRD analysis can be used to determine the chemical composition of composite materials. Generally speaking, the hydration products of cement include C-S-H gel, Ca(OH)_2_, and sulphoaluminate hydrates (Aft and AFm). XRD analysis of normal cement slurry shows that the hydration products usually exist as phases such as Ca(OH)_2_, CaCO_3_, and other types of C-S-H gel. Using XRD, we investigated the influence of different BFS-CFFA ratios on the chemical composition of cementless concrete specimens. XRD analysis presents different peak values versus scattering angle as known chemical compounds (hydrations). Figure 11 compares the XRD patterns of the specimens at 28 days. As can be seen, the Ca(OH)_2_, CaCO_3_, and Ca_3_SiO_5_ contents of the cementless specimens do not change drastically, but the cementless specimens display stronger CaSO_4_ peaks than the control groups. In addition, the more BFS and less CFFA a specimen contains, the weaker its CaO and SiO_2_ peaks are. Peaks for 2CaO-Ai_2_O_3_-SiO_2_ also appeared at 31.5°. This shows that mixing CFFA and BFS together to create cementless concrete can effectively stimulate the potential gel properties of the BFS and generate more calcium silicon oxides, which facilitate the development of C-S-H gel, enhance compactness, and effectively increase concrete strength.

## 4. Conclusions

This study examined the influence of completely replacing cement with CFFA and BFS on the fresh concrete properties, physical properties, mechanical properties, microscopic properties, and durability properties of pervious concrete. Two major factors were considered: the ratio of water-quenched BFS and CFFA from CFBC used to replace cement and the FPV of the pervious concrete. The strong alkaline characteristics of the CFFA served as the alkaline activator of the BFS. In the various specimens, we replaced 50%, 60%, and 70% of the weight of the cement with BFA and the remaining 50%, 40%, and 30% with CFFA as the alkaline activator. The performance of the resulting specimens was compared with that of pervious concrete specimens made with pure cement. Furthermore, we also examined the influence of different FPVs on pervious concrete properties. Based on the test results, we arrived at the following conclusions:

Replacing cement with CFFA and BFS in pervious concrete

1. Replacing cement with CFFA and BFS can improve the bleeding of the specimens and shorten the setting time but it reduces workability.
2. Replacing cement with CFFA and BFS results in lower compressive strength than that in pervious concrete made with cement (only 90% of that after 28 days of curing). However, for normal engineering applications, such concrete still has adequate strength to serve as the materials of waterworks structures or pervious backfill materials that do not need to be compacted, which is 1.5 MPa to 14 MPa.
3. Replacing cement with CFFA and BFS gives pervious concrete greater resistance against chemical substances. However, as the proportion of CFFA increases, the concrete’s resistance to chloride ion penetration declines, which affects the durability of the concrete. Thus, in terms of chemical resistance, a BFS:CFFA ratio of 7:3 is optimal, and the resulting chemical resistance was even better than that of cement specimens in the control groups. What is worth noting is that the chemical resistance remains good even when the FPV is reduced. Whether the FPV can be reduced further for better permeability is an issue worth investigating in the future.
4. From the perspective of sustainable development, cement manufacturing today is accompanied by massive greenhouse gas emissions. Manufacturing 1 ton of cement means releasing 1 ton of carbon dioxide. Fly ash is a by-product of coal burning, and recycling it will help to achieve energy conservation and reduce carbon emissions and air pollution, which has a positive impact on the environment. At present, there are many similar applications employing CFFA in construction materials around the world (such as controlled low-strength materials).

Different FPVs in pervious concrete

1. A higher FPV means greater compactness and unit weight in the pervious concrete. Compressive and splitting strength tests indicated slightly poorer strength in specimens with a lower FPV. However, the normal cement specimens and the specimens with BFS:CFFA = 5:5 presented significant differences in permeability and chemical resistance, so more attention will need to be paid to the FPV in pervious concrete in the future.
2. The water absorption rate test revealed higher water-absorbing capabilities in specimens with a higher FPV. Thus, it is possible that the pores in the pervious concrete specimens became filled with gel, which increased the water absorption rate.
3. The cementless pervious concrete specimens with BFS:CFFA = 7:3 and FPV = 90% presented better compressive strength, permeability, and resistance to chemical corrosion.

## Figures and Tables

**Table 1:** Physical and chemical properties of cement and by-product materials.

Materials	Physical Test						Chemical Analysis						
	Fineness by Air Permeability (m^2^/kg)	Amount Retained on #325 Sieve (%)	Density (g/cm^3^)	Activity Index		Air Content of Mortar (%)	Loss on Ignition (%)	SiO_2_ (%)	Al_2_O_3_ (%)	Fe_2_O_3_ (%)	CaO (%)	MgO (%)	SO_3_ (%)
				7 Days (%)	28 Days (%)								
Cement	345	-	3.15	-	-	7.2	1.75	20.42	4.95	3.09	61.96	3.29	2.4
CFFA	-	-	2.73	92	99	-	-	29.47	19.27	3.49	35.54	1.82	7.36
BFS	586	0.7	2.88	112	133	3.65	0.1	33.68	14.37	0.29	40.24	7.83	0.66

**Table 2:** Mix designs of pervious concrete (kg/m^3^).

Mix No.	W/C	Water	Cement	BFS	CFFA	Coarse Aggregates	Superplasticizer	Thickener
5A	0.35	120.38	-	207.54	207.54	1619.00	24.91	2.08
5B	0.35	137.58	-	237.21	237.21	1619.00	28.47	2.37
5C	0.35	154.79	-	266.87	266.87	1619.00	32.03	2.67
6A	0.35	132.83	-	249.05	166.03	1619.00	12.45	2.08
6B	0.35	151.82	-	284.66	189.77	1619.00	14.23	2.37
6C	0.35	170.80	-	320.25	213.50	1619.00	16.01	2.67
7A	0.35	145.28	-	290.56	124.53	1619.00	0.00	2.08
7B	0.35	166.05	-	332.10	142.33	1619.00	0.00	2.37
7C	0.35	186.81	-	373.62	160.12	1619.00	0.00	2.67
CA	0.35	145.28	415.07	-	-	1619.00	0.00	0.00
CB	0.35	166.05	474.43	-	-	1619.00	0.00	0.00
CC	0.35	186.81	533.74	-	-	1619.00	0.00	0.00
